# Context-dependent trait covariances: how plasticity shapes behavioral syndromes

**DOI:** 10.1093/beheco/araa115

**Published:** 2020-11-17

**Authors:** David J Mitchell, Thomas M Houslay

**Affiliations:** 1 Department of Zoology/Ethology, Stockholm University, Svante Arrheniusväg 18B., Stockholm, Sweden; 2 Department of Zoology, University of Cambridge, Cambridge, UK

**Keywords:** animal personality, individual-by-environment interactions, mixed effect models, phenotypic integration, temporal plasticity

## Abstract

The study of behavioral syndromes aims to understand among-individual correlations of behavior, yielding insights into the ecological factors and proximate constraints that shape behavior. In parallel, interest has been growing in behavioral plasticity, with results commonly showing that animals vary in their behavioral response to environmental change. These two phenomena are inextricably linked—behavioral syndromes describe cross-trait or cross-context correlations, while variation in behavioral plasticity describes variation in response to changing context. However, they are often discussed separately, with plasticity analyses typically considering a single trait (univariate) across environments, while behavioral trait correlations are studied as multiple traits (multivariate) under one environmental context. Here, we argue that such separation represents a missed opportunity to integrate these concepts. Through observations of multiple traits while manipulating environmental conditions, we can quantify how the environment shapes behavioral correlations, thus quantifying how phenotypes are differentially constrained or integrated under different environmental conditions. Two analytical options exist which enable us to evaluate the context dependence of behavioral syndromes—multivariate reaction norms and character state models. These models are largely two sides of the same coin, but through careful interpretation we can use either to shift our focus to test how the contextual environment shapes trait covariances.

## Teaser

How can consistent individual differences in behavior be maintained across separate traits, while the same behavior may not be consistent across environmental conditions? Here, we show how we can test for context dependence of behavioral syndromes. By quantifying multiple behaviors while simultaneously manipulating conditions, we can evaluate how environments affect behavioral trait covariances.

Much recent effort has focused on among-individual variation in behavior (animal “personality”), with particular regards to understanding the ecological factors (Bell and Sih 2007; Dingemanse et al. 2009; Adriaenssens and Johnsson 2013) and proximate constraints (Biro et al. 2014), which promote these trait covariances. These covariances have been termed “behavioral syndromes,” with the initial definition encompassing correlations across both traits (e.g., boldness and activity correlations) and contexts (e.g., foraging activity in different habitats) (Sih et al. 2004). Over time, the working definition of behavioral syndromes has narrowed to describing a suite of correlated behavioral traits (Dingemanse and Wright 2020; Hertel et al. 2020), while the evaluation of cross-context behavioral correlations is typically studied separately as individual variation in plasticity (Martin and Réale 2008; Dingemanse et al. 2010). This separation occurs despite variation in contextual (A.K.A. “activational”) plasticity being central to the idea of syndromes, as it implies that individual differences in behavior are not fully maintained across environments (Brommer 2013). This further implies that correlations among traits are themselves not consistent across environmental conditions (Stearns et al. 1991).

Here, we argue that an understanding of how the contextual environment shapes trait correlations will help reintegrate behavioral syndromes and plasticity variation. The environment is known to shape trait covariances over different timescales, with correlational selection leading to adaptive trait covariances (i.e., integration) across generations (Lande and Arnold 1983; Dingemanse and Réale 2005). Developmental plasticity can promote behavioral syndromes during early ontogeny (Bell and Sih 2007; Adriaenssens and Johnsson 2013), which may point to a genotype-by-environment interaction (GxE) architecture that promotes favored trait combinations in certain environments or reflect trade-offs among traits. These effects of developmental plasticity are usually inferred post hoc through group comparisons of individuals previously exposed to different stimuli (Stamps 2016). Here, we show that a similar perspective can be taken across the contextual environment, whereby individual variation in plasticity (individual-by-environment interaction, or IxE) can alter the strength—or appearance—of behavioral correlations.

Studies of individual variation in plasticity have typically looked at how changes in environmental contexts affect a single aspect of animal behavior. In contrast, behavioral correlations are typically investigated within a single environmental context. Even when data are available to model plasticity in multivariate behavior, studies often present a series of univariate tests for variation in behavioral plasticity (e.g., Biro et al. 2010; Urszán Tamás et al. 2018), or model and present tests for trait correlations and plasticity separately (e.g., Hertel et al. 2020). Recent efforts to merge these topics have taken a perspective of plasticity as an extension of personality variation. Studies now show individual variation in behavioral plasticity may itself be repeatable or heritable (Araya-Ajoy and Dingemanse 2017; Mitchell and Biro 2017) and correlated with mean behavior (e.g., Carter et al. 2012, but see Box 1). Recent papers have even looked for “plasticity syndromes” (Dingemanse and Wolf 2013), quantifying the correlations in responsiveness of the same behavior to multiple environmental factors (Mitchell and Biro 2017; Cornwell et al. 2019) and testing whether plasticity in separate traits is correlated (Saltz et al. 2017; Gibelli et al. 2018).

A potential danger of focusing too heavily on plasticity is that such research tends to center on the magnitude of change in trait expression. Discussions of variation in plasticity often lean on predictions of limitations of constraints to plasticity (DeWitt et al. 1998), with individuals being limited by their condition or the availability of information to achieve an assumed (but usually unknown) optimal behavior (Dubois 2019). From this perspective, cross-context correlations in a behavioral trait arise through “behavioral carryovers” caused by constraints on plasticity (Sih et al. 2004). However, this sets the assumption that plasticity is under selection, rather than the trait value expressed in a given environment. This view may often not be appropriate, as individuals within the same population and responding to the same stimulus commonly vary not just in the magnitude of plasticity (absolute value of the reaction norm slope), but also the sign of change (positive and negative slopes). Therefore, individuals on both ends of the spectrum of change can be considered “plastic” (Stamps 2016). It seems strange then to assume animals that showed no plastic change were “constrained” when in fact their response was close to the mean response—but this line of reasoning has led researchers to focus on the absolute slope (e.g., Gibelli et al. 2018; Barou-Dagues et al. 2020).

As environments change, selection pressures change—thus, animals change trait expression to match the new environment. However, rather than assume a shift in trait optima, we may be better assuming a shifting fitness landscape that favors different trait combinations and thus individual-specific trait optima. In such a scenario, it would provide a clearer and more parsimonious view of plasticity to focus on behavioral trait expression under a given environmental state (Via and Lande 1985). This requires a shift in focus in our analyses, and we can instead choose to analyze a single behavior in two different contexts (e.g., risk vs. no risk, summer vs. winter) as separate but potentially correlated traits (i.e., cross-context correlation). This approach is known as the “character state” model and sets a correlation of 1 across environments, and no change in environment-specific variances, as the null hypothesis of no plasticity variance. This can easily be scaled up to multiple traits, at which point we can compare environment-specific covariance matrices (e.g., Roff et al. 2012).

This approach was recently used to test for individual variation in a suite of movement behaviors among guppies (*Poecilia reticulata*) responding to different forms of predation risk (Houslay et al. 2018). There were strong population-level responses to the simulated predation risk, and evidence for among-individual variation in plasticity, indicated by cross-environmental correlations as low as 0.42. Despite this plasticity variance, behavioral trait (co)variances in the absence of risk were largely maintained in the presence of risk. Of course, this conservation of behavioral (co)variance structure across contexts in the face of plasticity variation will not always be the case. In a study of blue tits (*Cyanistes caerulues*), Class and Brommer’s (2015) found a behavioral syndrome of docility and handling-induced stress (measured as breathing rate) was present in nestlings but not adults, demonstrating clearly the need for correlations between labile traits to be assessed across different contexts.

While character state models are a particularly useful tool when estimating plasticity across discrete environments, they can quickly become unwieldy and quantitatively heavy when the environment is continuous. This is because they fit separate population means and individual specific predictions for each environmental state. In the case of continuous environmental predictors, a trait can instead be modeled as a function of the environment—that is, the more familiar reaction norm approach (Falconer 1981; Roff 1997), which has been the dominant analytical tool of behavioral ecologists (Dingemanse et al. 2010). Here, non-zero variance in the slopes of the reaction norms provides evidence for plasticity variance. The reaction norm approach provides a more intuitive description of phenotypic plasticity in a single trait (a line as a function of the environment, rather than distinct points in state space), but comes with additional assumptions of the shape of the relationship between the environment and trait (normally linearity).

When linear reaction norms are scaled up to multiple traits we can quantify all the among-individual correlations between intercepts and slopes, though interpretation can be tricky. Slope–slope correlations, for example, are informative but need to be placed in the context of the other model parameters. In Box 1, we provide and demonstrate how multivariate reaction norm and character state models can be used to test the consistency of behavioral syndromes across environmental contexts (and how to derive one from the other). In this simple two-context scenario, these models are mathematical equivalencies. As complexity increases, the choice of analytical framework may be driven by experimental or sampling design. Reaction norms may commonly follow nonlinear patterns (e.g., performance curves), in which case nonlinear reaction norms can also be specified (Morrissey and Liefting 2016). Other instances may predict punctuated change, such as at metamorphosis or sexual maturity, and here an equation may not be easily defined. Character state models are more appropriate when there are more than two environments but these cannot be placed on a continuous axis. In controlled situations, assumptions of linearity can be relaxed through character state models of a small number of sample environments, which we demonstrate in the supplements with an example of a thermal performance curve (Mitchell and Houslay 2020). While the modeling framework may often be driven by practical constraints, it is helpful to consider the complementary nature of these approaches.

**BOX 1: ASSESSING SYNDROME STABILITY FROM MULTIVARIATE REACTION NORMS**

When considering two discrete environments, character state and reaction norms models are mathematically equivalent, although estimate different parameters and therefore emphasize different biology. Using the reaction norm framework, it can be tempting to consider the intercept and slope as distinct but correlated processes (i.e., “personality” and “plasticity”). The character state model tends to emphasis variation in behavioral trait values within each environment, and the correlation between them. We believe that an understanding of both analytical frameworks, and their complementary nature, is important to maintaining a focus on the biological processes they describe.

**Figure 1:**
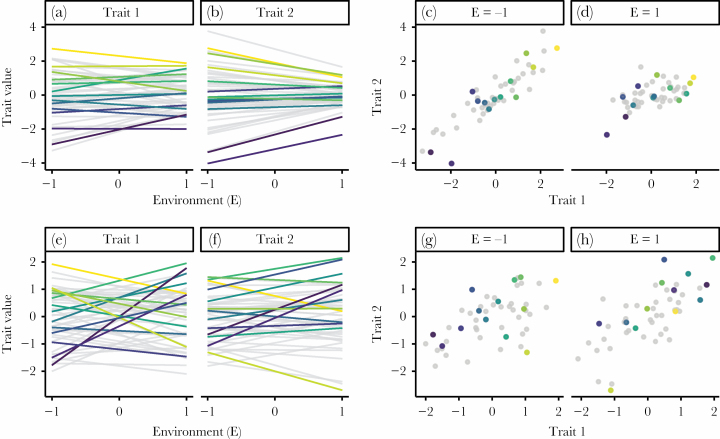
Displayed are the reaction norms and character state plots of two distinct scenarios (upper row, a–d, and lower row, e–h). Reaction norms (a–b, e–f) illustrate individual variation in plasticity across an environmental gradient from −1 to 1, separately for traits 1 and 2. Scatterplots illustrate the bivariate relationship between traits 1 and 2 at *x =* −1 and *x =* 1. A subset of individuals are colored for illustrative purposes, with colors corresponding to a scaling of trait 1 values at *x =* −1 within each scenario (a, e). As environments change from left to right in the reaction norm plots, predicted values of individuals change for both traits within each scenario. This means the location of individuals in the scatterplots change from *x =* −1 to *1*. At *x =* −1, the color gradient is clearly shown along the *x*-axis (c,g). On the top, the cross-trait intercept–slope and slope–slope covariances lead to the strong positive covariance seen at *x =* −1 (c) disappearing at *x =* 1 (d), while the color gradient is also reduced. In contrast, the shape of the covariance is maintained on the bottom from *x =* −1 (g) to *x =* 1 (h); however, due to reaction norm variance the location of individuals within this covariance is shuffled, leading to the reduced color gradient.

Quantifying the extent to which behavioral covariances are affected by environments requires collecting data on multiple traits with two or more repeated measures under each environment. A bivariate reaction norm model is given by Equation 1, with the predicted mean (or “fixed” intercept) behavior at *x = 0* given by *β*_*-,0*_ and the mean slope given by *β*_*-,1*_. Estimates for each individual (*ID*) are then given as a deviance from the *β* estimates, with predictions assumed to be multivariate-normal. Together, Equation 1 describes the predictions of Figure 1, and yields a four-by-four covariance matrix at the individual level (Equation 2), with $\sigma_{-,-}^{2}$ being variances. Through these estimates, we can calculate cross-environmental correlations in a trait (Roff 1997; Falconer 1981; Brommer 2013) and the changes in covariances between traits (Brommer and Class 2015). First, among-individual variance in the predicted mean behavior (“*Pred*”) changes as a function of the environment (i.e., “*x*”), and is given by Equation 3. When the environment is continuous, non-zero slope variance means behavioral variance changes, with an intercept–slope *cov ≥ 0* leading to fanning and *cov < 0* leading to convergence as *x* increases. Thus, even when *cov = 0*, this implies an important relationship between the intercepts and slopes. This covariance also changes with contrasting placement of the intercept, following the equations $cov_{Pred,slope}[x]=cov_{int,slope}+\;\sigma_{0,1}^{2}x$. Second, the cross-environmental context covariance in a single behavior is given by Equation 4. Together with variances calculated from Equation 3, this can be converted to a correlation coefficient (*r*). When $\sigma_{slope}^{2}>0$ in a reaction norm model, *r* < 1 in a character state model (unless the intercept–slope covariance leads to perfect fanning or convergence). Finally, the covariance between different behavioral traits as a function of environmental context is given by Equation 5. Notably, the “*x*” predictors for the two response variables can be specified independently, meaning one could calculate correlations of “Trait 1” and “Trait 2” at different environmental values (e.g., unhabituated open-field test to aggression in a familiar environment). Alternatively, the two traits may be responding to different environmental gradients. Taken together, these equations demonstrate how behavioral syndromes change as a function of the environment when plasticity varies in either trait. In the Supplementary Material, we break down these equations further (Mitchell and Houslay 2020).

$$\begin{array}{l} \begin{array}{l} y_{1}\\ y_{2} \end{array}∼\begin{array}{l} \left(\beta_{1,0}+ID_{1,0}\right)+\left(\beta_{1,1}+ID_{1,1}\right)x\\ \left(\beta_{2,0}+ID_{2,0}\right)+\left(\beta_{2,1}+ID_{2,1}\right)x \end{array} \end{array}$$ (1)

$$\begin{array}{l} \begin{array}{l} \begin{array}{l} ID_{1,0}\\ ID_{1,1} \end{array}\\ \begin{array}{l} ID_{2,0}\\ ID_{2,1} \end{array} \end{array}=MVN\left(0,\;\;\;\begin{array}{llll} \sigma_{1,0}^{2} & & & \\ cov_{a} & \;\sigma_{1,1}^{2} & & \\ cov_{b} & \;cov_{c} & \;\sigma_{2,0}^{2} & \\ cov_{d} & \;cov_{e} & \;cov_{f} & \;\sigma_{2,1}^{2} \end{array}\right) \end{array}$$ (2)

$$\begin{array}{l} \sigma_{Pred[x]}^{2}.\;\;\;=\;\;\;\sigma_{1,0}^{2}+2cov_{a}x+\sigma_{1,1}^{2}x^{2} \end{array}$$ (3)

$$\begin{array}{l} cov_{E[x_{1}],E[x_{2}]}=\;\;\;\sigma_{1,0}^{2}+cov_{a}x_{1}+cov_{a}x_{2}+\sigma_{1,1}^{2}x_{1}x_{2} \end{array}$$ (4)

$$\begin{array}{l} cov_{Pred[x1],Pred[x2]}.\;\;\;=\;\;\;cov_{b}+cov_{c}x_{1}+cov_{d}x_{2}+cov_{e}x_{1}x_{2} \end{array}$$ (5)

This relationship between plasticity and trait correlations has been largely obfuscated among behaviorists. Analyses in behavioral ecology typically mean-center environmental predictors and treat the intercept as “personality” and the slope as “plasticity” (Dingemanse et al. 2010). This is convenient in that it estimates the grand-mean behavior of individuals, allowing cross-trait correlations of this mean. However, as we demonstrate above, this cross-trait correlation cannot be generalized to other sampled environments and further might not always be the most biologically meaningful environmental context. For instance, in a novel object test the first assay (prior to any habituation) may be the best measure of boldness (Frost et al. 2007). Thus, time should be left-centered, rather than mean-centered. Aggression may be most relevant during the breeding season, so we may wish to center environmental predictors to reflect the conditions of this season. It therefore seems intuitive that not only does plasticity variation mathematically infer changes in behavioral trait covariances across environments, existing theory often predicts these changes—there is no reason to predict latency to inspect a no longer novel object should covary with another metric of boldness (e.g., flight initiation distance).

Whether using character state or reaction norm models, or deriving one from the other, it is important to consider all (co)variances that together describe patterns of changes in behavior. This broadening of focus de-emphasizes differences in plasticity and emphasizes differences in mean behavior across environments, attempting to understand the behavioral trait expression in that environmental context (Via and Lande 1985). Different changes in covariance will naturally have different biological implications, and researchers must carefully consider the appropriate null hypotheses. First, where traits are directly linked (e.g., activity and routine metabolic rate), we should expect stability of the syndrome across environmental conditions. Variances may expand or contract, and the location of specific individuals in the trait space may change, but the covariance will stay similar (Figure 1 lower)—as was observed in the movement behaviors of guppies (Houslay et al. 2018). This could also be stated as a correlation of slopes across the two traits, as was observed in a study of standard and maximal metabolic rates (Careau et al. 2014). In this case, the null hypothesis to be tested is not that the slopes are uncorrelated (*r*_slope,slope_ = 0), but rather that they are perfectly correlated (*r*_slope,slope_ = +1 or −1). Such effects would also predict a residual covariance congruent with the intercept–intercept and slope–slope covariances. Second, correlations may be predicted to change as a function of the environment (Figure 1 upper), for instance, if animals are switched from ad libitum to restricted feeding, we may expect a switch from no correlation or a performance model to an allocation model, shown by a negative covariances between costly traits (King et al. 2011). Stated in terms of reaction norms, this is a combination of all cross-trait covariances (Box 1).

This is not to question the validity or importance of the explicit focus on plasticity where appropriate; under certain situations, a focus on the magnitude (or speed) of change does make sense, for example, in understanding variation in learning (Buechel et al. 2018; Madden et al. 2018). This can be particularly interesting where efforts are taken to standardize potential confounds, such as motivation, to assess ability to exhibit plastic change rather than realized change. Predictions derived from constraints to plasticity are valuable: there are known and important costs of maintaining the ability to perceive and compute environmental information (e.g., the “expensive brain” Kotrschal et al. 2013), which speak to stimulus–response relationships (i.e., reaction norms). However, this needs to be complemented by an understanding of the fitness consequences of the expressed behavior and relationship to the broader phenotype under different contexts. A view of plasticity as a separate but potentially correlated trait may lead to tunnel vision and inhibit our understanding of multivariate behavioral variation. Large bodies of literature now show that traits do not operate independently, meaning the environment shapes trait covariances across generations through evolutionary change, and within generations through developmental plasticity. It seems only logical that this phenomenon should extend to the contextual environment.

## Supplementary Material

araa115_suppl_Supplementary_Material
