# Supplementary material for: Context-dependent trait covariances: how plasticity shapes behavioral syndromes
Source: Behav Ecol. 2020 Nov 17;32(1):25–9. doi: 10.1093/beheco/araa115 (PMC7937033; doi:10.1093/beheco/araa115)
Supplement: araa115_suppl_Supplementary_Material [file araa115_suppl_supplementary_material.docx]

**Supplementary material**

**Part 1: One behaviour in two environments**

We will start by demonstrating the simplest scenario – one trait across two contexts. This may be risk vs. no risk, low vs. high temperature, day vs. night etc. In this simple example, where you have repeated measures of a sample of individuals in both contexts, this could be modelled using either (i) reaction norm with the equation $y\sim\left( \beta_{0}+ID_{0} \right)+\left( \beta_{1}+ID_{1} \right)x+ \varepsilon$, where the subscripts refer to intercept (0) and linear slope (1), which yields this among-individual covariance matrix; $ID=BVN\left( 0,\left[ \begin{matrix} ID_{0} & \\ cov_{0,1} & ID_{1} \end{matrix} \right] \right)$. Or (ii) this can be fitted as a character state model with the equation $\begin{matrix} y_{1} \\ y_{2} \end{matrix}\sim\begin{matrix} \mu_{1}+ID_{\mu1}+\varepsilon_{1} \\ \mu_{2}+ID_{\mu2}+\varepsilon_{2} \end{matrix}$, where the subscripts refer to the distinct environments. This model yields this matrix; $ID=BVN\left( 0,\left[ \begin{matrix} ID_{\mu_{1}} & \\ cov_{\mu_{1},\mu_{2}} & ID_{\mu_{2}} \end{matrix} \right] \right)$. These models are analogous, except for the one subtle difference that the character state is fitting separate residual variances for the two environments, though this can be changed. Sticking initially with a reaction norm model, we may get something like this:


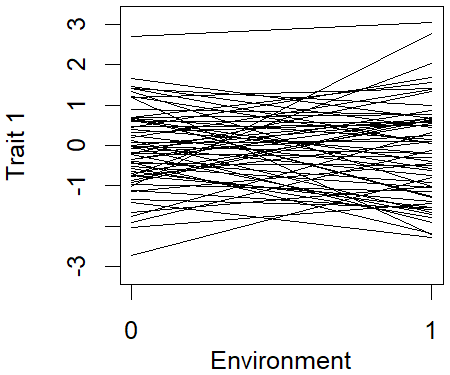


These random intercepts and slopes follow a bivariate normal distribution; $ID=BVN\left( 0,\left[ \begin{matrix} 1.1 & \\ -0.7 & 1.4 \end{matrix} \right] \right)$. This represents deviances from the fixed effects, so the mean is 0. We then have the variance covariance matrix, giving an among-individual variance at the intercept (*E = 0*) of 1.1 and variance in the slopes of 1.4. These two factors covary at -0.7, or a correlation coefficient of *r* = -0.56 (note that the covariance matrix is symmetric around the diagonal, so we have only included below-the diagonal). This covariance could also be shown as a scatterplot, appearing like this:


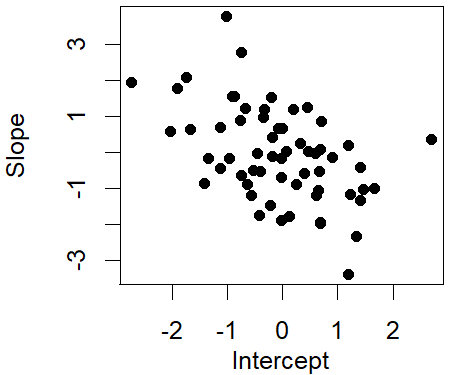


This can be converted to a character state covariance matrix with the formula:

$$\left[ \begin{matrix} \sigma_{E1}^{2} & cov\left[ E_{1},E_{2} \right] \\ cov\left[ E_{1},E_{2} \right] & \sigma_{E2}^{2} \end{matrix} \right]=\left[ \begin{matrix} 1 & E_{1} \\ 1 & E_{2} \end{matrix} \right]*\left[ \begin{matrix} \sigma_{int}^{2} & cov_{a} \\ cov_{a} & \sigma_{slp}^{2} \end{matrix} \right]*\left[ \begin{matrix} 1 & 1 \\ E_{1} & E_{2} \end{matrix} \right]$$

Where *E*_1_ and *E*_2_ are different values of the environmental predictor. For those like me who struggle with matrix algebra, this works out to be:

$$\left[ \ldots\right]=\left[ \begin{matrix} \sigma_{int}^{2}+2cov_{a}E_{1}+ \sigma_{slp}^{2}E_{1}^{2} & \sigma_{int}^{2}+cov_{a}E_{1}+cov_{a}E_{2}+ \sigma_{slp}^{2}E_{1}E_{2} \\ \sigma_{int}^{2}+cov_{a}E_{1}+cov_{a}E_{2}+ \sigma_{slp}^{2}E_{1}E_{2} & \sigma_{int}^{2}+2cov_{a}E_{2}+ \sigma_{slp}^{2}E_{2}^{2} \end{matrix} \right]$$

In the simple 2 environment example where $E_{1}=0$ and $E_{2}=1$, this yields the character state matrix – i.e. $\left[ \begin{matrix} ID_{\mu_{1}} & \\ cov_{\mu_{1},\mu_{2}} & ID_{\mu_{2}} \end{matrix} \right]$. In this instance, the equations simplify to:

$$\left[ \ldots\right]=\left[ \begin{matrix} \sigma_{int}^{2} & \sigma_{int}^{2}+cov_{a} \\ \sigma_{int}^{2}+cov_{a} & \sigma_{int}^{2}+2cov_{a}+ \sigma_{slp}^{2} \end{matrix} \right]$$

Through substituting the values from the reaction norm matrix into these equations, we can calculate the change in variance from the left ($E_{1}=0$) to the right ($E_{2}=1$) as ‘$1.1+2*\left( -0.7 \right)+1.4$’ which equals 1.1. In this example, the variance did not change, as we can see in the initial plot. However, we can clearly see that lines are crossing and therefore the trait differences are not fully maintained across the x-axis. A cross-environment correlation of this trait looks like this:


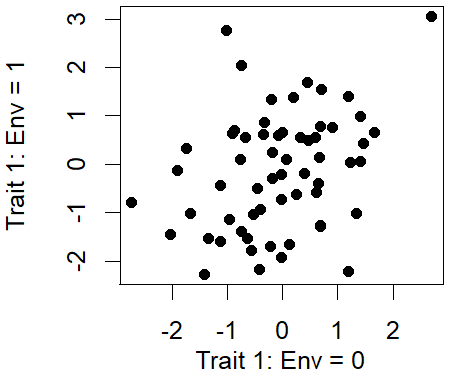


So, we clearly have a correlation that is less than 1 (due to the variance in plasticity), but also greater than 0 – indicating differences are partially maintained. But, to what extent are they maintained? We can use the bottom left cell above (Eq. 4 in the main text): $1.1+\left( -0.7 \right)$, which equals a covariance of 0.4. We could convert this into a correlation coefficient: $r=\frac{0.4}{sqrt(1.1*1.1)}=0.36$.

**Two behavioural traits**

Now let’s add a second behaviour, so we have two sets of reaction norms. For the second behaviour (‘*z*’), individual variation is described by the covariance matrix in the reaction norm model as $ID=BVN\left( 0,\left[ \begin{matrix} 0.9 & \\ -0.5 & 1 \end{matrix} \right] \right)$. Going through the same steps as above, we can get the character state matrix to see that again the variance does not change and there remains a positive covariance; $BVN\left( 0,\left[ \begin{matrix} 0.9 & \\ 0.4 & 0.9 \end{matrix} \right] \right)$ .


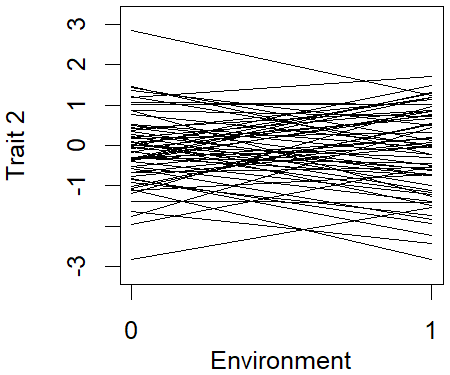


Now, to bring these two scenarios into multivariate reaction norms, we must add all the cross-trait covariances among individuals: intercept(*y*)-intercept(*z*) (*cov­_b_*), intercept(*y*)-slope(*z*) (*cov_d_*), intercept(*z*)-slope(*y*) (*cov_c_*), slope(*y*)-slope(*z*) (*cov_e_*). As we showed in the main text, this reaction norm covariance matrix looks like this:

$MVN\left( 0,\left[ \begin{matrix} \sigma_{y,0}^{2} & & & \\ cov_{a} & \sigma_{y,1}^{2} & & \\ \boldsymbol{co}\boldsymbol{v}_{\boldsymbol{b}} & \boldsymbol{co}\boldsymbol{v}_{\boldsymbol{c}} & \sigma_{z,0}^{2} & \\ \boldsymbol{co}\boldsymbol{v}_{\boldsymbol{d}} & \boldsymbol{co}\boldsymbol{v}_{\boldsymbol{e}} & cov_{f} & \sigma_{z,1}^{2} \end{matrix} \right] \right)$

The equivalent character state model matrix for the two-context environment would look like this:

$MVN\left( 0,\left[ \begin{matrix} \sigma_{\mu_{y1}}^{2} & & & \\ cov_{\mu_{y1},\mu_{y2}} & \sigma_{\mu_{y2}}^{2} & & \\ cov_{\mu_{y1},\mu_{z1}} & cov_{\mu_{y2},\mu_{z1}} & \sigma_{\mu_{z1}}^{2} & \\ cov_{\mu_{y1},\mu_{z2}} & cov_{\mu_{y2},\mu_{z2}} & cov_{\mu_{z1},\mu_{z2}} & \sigma_{\mu_{z2}}^{2} \end{matrix} \right] \right)$

The equations from the single trait section hold for the top 2-by-2 segment (*y*) and the bottom right 2-by-2 segments (*z*) of the reaction norm matrix, which each describe one of the two traits. Conveniently, this also holds for the cross-trait segment in bold, which describes all cross-trait covariances:

$$\left[ \begin{matrix} cov_{Pred_{y}\left[ E_{1} \right], Pred_{z}[E_{1}]} & cov_{Pred_{y}\left[ E_{2} \right], Pred_{z}[E_{1}]} \\ cov_{Pred_{y}\left[ E_{1} \right], Pred2[E_{2}]} & cov_{Pred_{y}\left[ E_{2} \right], Pred_{z}[E_{2}]} \end{matrix} \right]=\left[ \begin{matrix} 1 & E_{1} \\ 1 & E_{2} \end{matrix} \right]*\left[ \begin{matrix} cov_{b} & cov_{c} \\ cov_{d} & cov_{e} \end{matrix} \right]*\left[ \begin{matrix} 1 & 1 \\ E_{1} & E_{2} \end{matrix} \right]$$

Which breaks down to:

$$\left[ \ldots\right]=\left[ \begin{matrix} cov_{b}+2cov_{c}E_{1}+cov_{e}E_{1}^{2} & cov_{b}+cov_{c}E_{2}+cov_{d}E_{1}+cov_{e}E_{1}E_{2} \\ cov_{b}+cov_{c}E_{1}+cov_{d}E_{2}+cov_{e}E_{1}E_{2} & cov_{b}+cov_{c}E_{2}+cov_{d}E_{2}+cov_{e}E_{2}^{2} \end{matrix} \right]$$

Again, as *E* is 0 or 1, this simplifies to:

$$\left[ \ldots\right]=\left[ \begin{matrix} cov_{b} & cov_{b}+cov_{c} \\ cov_{b}+cov_{d} & cov_{b}+cov_{c}+cov_{d}+cov_{e} \end{matrix} \right]$$

In this instance, the simulated individual variance in reaction norms has this structure:

$MVN\left( 0, \left[ \begin{matrix} 1.1 & & & \\ -0.7 & 1.4 & & \\ 0.6 & -0.2 & 0.9 & \\ -0.5 & -0.1 & -0.5 & 1 \end{matrix} \right] \right)$

In the top left, we can see the segment of the matrix we started with (reaction norms for trait 1), and at the bottom right, the segment of the matrix for trait 2. The intercept at $E_{1}=0$ is 0.6 (the intercept-intercept covariance, *cov­_b_*), indicating the traits covary in the reference environment.


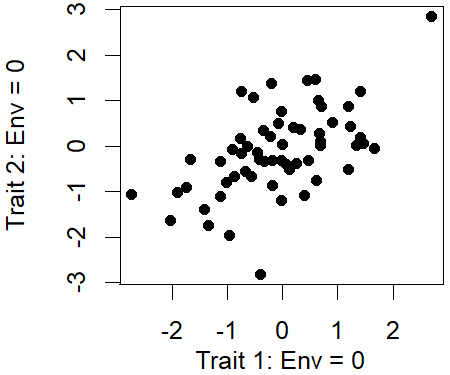


We also find negative cross-trait covariances between intercepts and slopes (intercept(y)-slope(*z*) (*cov_d_*) = -0.5; intercept(*z*)-slope(*y*) (*cov_c_*) = -0.2) and a weak slope-slope covariance (*cov_e_*) of -0.1.


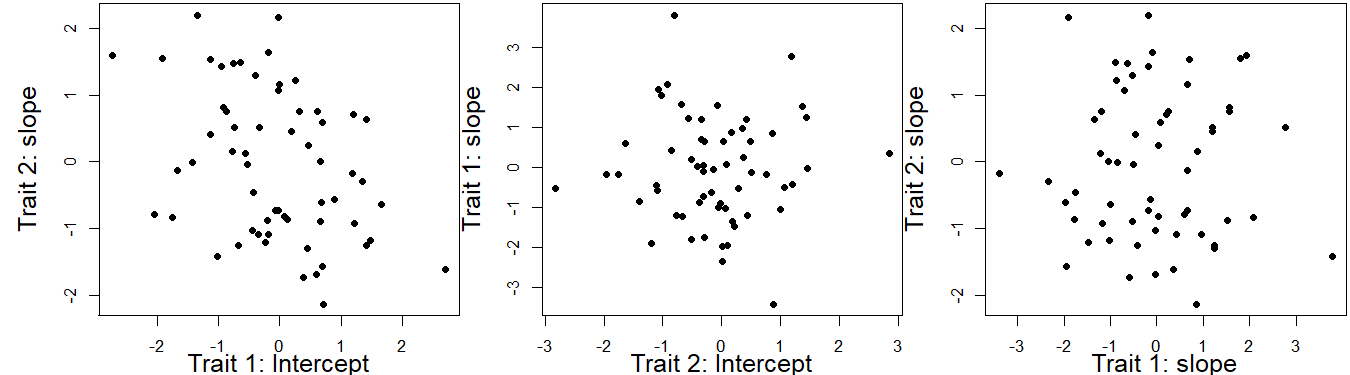


By substituting these numbers into the matrix above, we can calculate the character state output:

$MVN\left( 0, \left[ \begin{matrix} 1.1 & & & \\ 0.4 & 1.1 & & \\ 0.6 & 0.4 & 0.9 & \\ 0.1 & 0 & 0.4 & 0.9 \end{matrix} \right] \right)$

Here the upper-left quadrant describes the (co)variance in trait 1 as expressed in the two distinct environments, the lower-right describes (co)variance in trait 2 as expressed in the same two environments, and the lower-left (and upper-right as the matrix is symmetrical around the diagonal) describes all cross-trait covariances. In this hypothetical case, the covariance between traits which was present at the intercept (*E = 0*) disappears in the manipulated environment (*E = 1*).


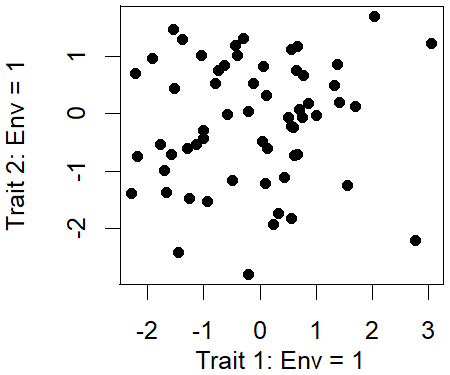


**Continuous reaction norms**

These equations are useful beyond the simple 2 context environments, in many cases we may wish to assess how plasticity across a continuous predictor affects trait covariations. For simplicity, what if we had an intermediate environment, so that *E_2_ = 0.5*, we could calculate the covariance between the two traits at E = 0.5 by substituting this value into this equations from above: $cov_{Pred_{y}\left[ E_{2} \right], Pred_{z}[E_{2}]}= cov_{b}+cov_{d}E_{2}+cov_{e}E_{2}^{2}=0.6+2*-0.2*0.5-0.1*{0.5}^{2}=0.225$. with *E_1_* from the equations above, giving:

$MVN\left( 0, \left[ \begin{matrix} 1.1 & & & \\ 0.75 & 0.75 & & \\ 0.6 & 0.5 & 0.9 & \\ 0.35 & 0.225 & 0.65 & 0.65 \end{matrix} \right] \right)$

At this midpoint, variance in both traits has now decreased, while the covariances have all changed slightly, with the exception of the intercept-intercept covariance (*E*_1_ *= 0*). Functions to implement these calculations from brms output can be found in here: (Mitchell, 2020) as supplementary material to (Mitchell et al., 2020).

**Part 2: Using character state models to model non-linear responses**

While reaction norm models can be extended to non-linear functions, these can quickly become difficult to interpret. Here we will show how the character state model could be useful in ameliorating assumptions of reaction norm models when the central tendency is non-linear. We’ll use a thermal performance curve, though non-linear change with environments is common throughout biology.

Thermal performance curves typically predict a gradual increase of the response variable in this case activity) to a maximum trait value with increasing temperature, then a steep decrease when temperatures are too hot. This looks something like the below curve. Collecting data across the full temperature range would be the best scenario, and ‘brms’ (Bürkner, 2017) and ‘nlme’ (Pinheiro et al., 2017) offer convenient specification of non-linear random regressions in the R environment. However, this is data intensive, and if one is interested in cross-context or cross-trait correlations, then these are no longer as simple to calculate as the linear reaction norm scenario in ‘Part 1’. Another option may be to take a focal subset of sample environments – which we illustrate using the coloured points below. At low temperatures (relative to the thermal peak), the change of the trait value with temperature is roughly linear and if the sample environments are within this range, linear reaction norms are a good approximation of the underlying biology (blue). Of course, one should not extrapolate beyond the data in this scenario, as this will quickly become misleading. Towards the peak, the relationship is highly curved and a linear reaction norm would do a poor job of explaining the central tendency (red). In this scenario, treating temperature as categorical may be beneficial. In many cases there will not be *a priori* knowledge of this curve, and it may be best to fit the model both ways and compare the fit.


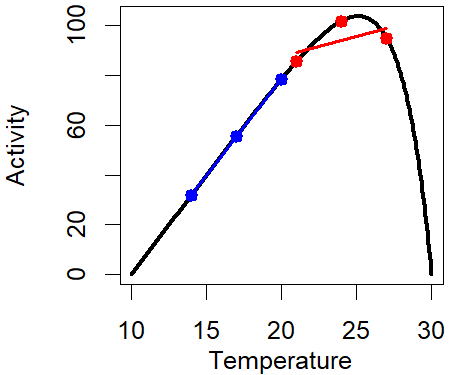


This curve is simply shown at the population level, however animals within a population are likely to vary in their peak activity rates, and the temperature range. For simplicity, we have simulated a scenario where animals i) vary in their activity rate at the peak of the curve, and ii) where the entire curve is shifted along the x-axis. The first creates a fanning pattern to the peak activity rate, the latter creates crossing of reaction norms and therefore inconsistency in their rank order of activity, i.e. personality differences are not fully maintained. From this, we’ll focus on three sample environments around the peak of the population curve, the red lines.


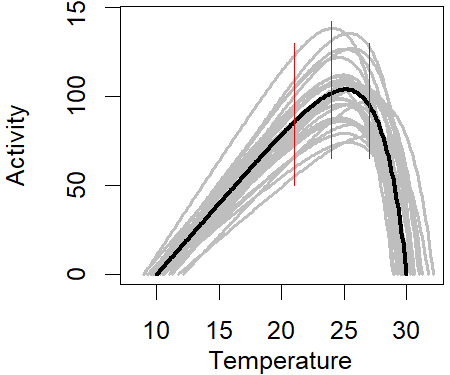


Quantifying this pattern effectively requires a minimum of 2 repeated measures for each individual in each environment, though more repeated measures is highly preferable. Two sample individuals are now highlighted showing ten repeated measures from their mean behaviour.


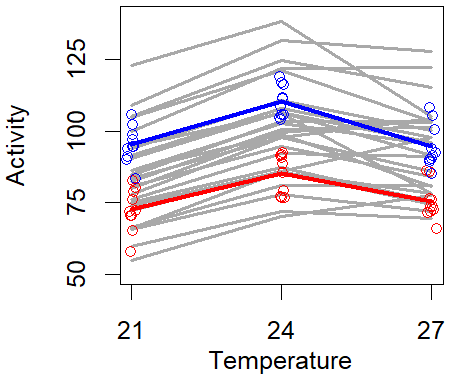


If only looking at one trait, these models can be fit in univariate packages by fitting a global intercept of 0 and temperature as a categorical variable. Thus, the model quantifies individual variances in means under each context and covariances between these means. On the random effects, we are now getting predicted values as deviances from that mean and covariances between these means. This model could be written as:

$$\begin{matrix} y_{21^{\circ}C} \\ y_{24^{\circ}C} \\ y_{27^{\circ}C} \end{matrix}\sim\begin{matrix} \mu_{21^{\circ}C}+{ID}_{21^{\circ}C} \\ \mu_{24^{\circ}C}+{ID}_{24^{\circ}C} \\ \mu_{27^{\circ}C}+{ID}_{27^{\circ}C} \end{matrix}+\varepsilon$$

With among individual (co)variances given as:

$$\begin{matrix} {ID}_{21^{\circ}C} \\ {ID}_{24^{\circ}C} \\ {ID}_{27^{\circ}C} \end{matrix}\sim MVN\left( 0, \begin{matrix} \sigma_{21^{\circ}C}^{2} & & \\ cov_{21^{\circ}C,24^{\circ}C} & \sigma_{24^{\circ}C}^{2} & \\ cov_{21^{\circ}C,27^{\circ}C} & cov_{24^{\circ}C,27^{\circ}C} & \sigma_{27^{\circ}C}^{2} \end{matrix} \right)$$

Again, consideration should be given to whether you assume equal variances at different temperatures. Together, this model yields the three covariances, which in the simulated case, correspond to these plots.


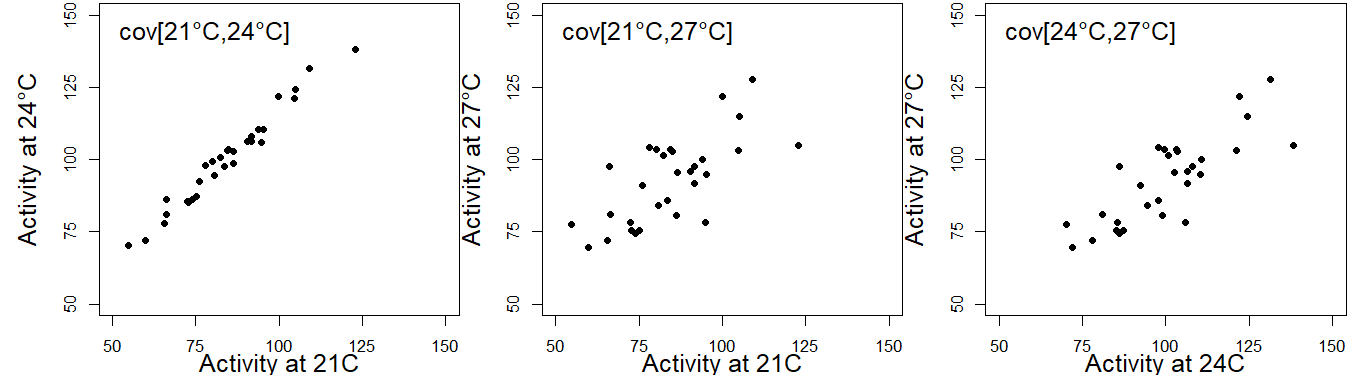


Finally, this model could easily be scaled up to a second trait to test predictions how traits may covary when under thermal stress or at thermal optima (e.g. due to phenotypic compensation). A second trait could then be measured under the three temperatures, which would yield a 6x6 covariance matrix, estimating covariances between traits and across temperatures. This could thus answer potential questions about trait integration in optimal vs. non-optimal temperatures.

Bürkner P-C, 2017. brms: An R package for Bayesian multilevel models using Stan. Journal of statistical software 80:1-28.

Mitchell DJ, 2020. Deakin guppy predation mesocosms. OSF.

Mitchell DJ, Beckmann C, Biro PA, 2020. Predation as a driver of behavioural variation and trait integration: effects on personality, plasticity, and predictability}. EcoEvoRxiv. doi: 10.32942/osf.io/jwd3c.

Pinheiro J, Bates D, DebRoy S, Sarkar D, 2017. {nlme}: Linear and Nonlinear Mixed Effects Models.
